# Acute otitis media in children: a vaccine-preventable disease?^☆^

**DOI:** 10.1016/j.bjorl.2017.02.004

**Published:** 2017-02-28

**Authors:** Marco Aurélio Palazzi Sáfadi, Daniel Jarovsky

**Affiliations:** a Santa Casa de São Paulo, Faculdade de Ciências Médicas, Departamento de Pediatria, São Paulo, SP, Brazil; b Hospital Infantil Sabará, Serviço de Infectologia Pediátrica, São Paulo, SP, Brazil

Acute otitis media (AOM) is one of the most common childhood diseases, being the main cause of medical consultations and antimicrobial prescriptions in developed countries, where infants receive an average of more than 40 days of antimicrobials per year. Prior to the introduction of pneumococcal conjugate vaccines, it was estimated that four of five infants would have an episode of AOM before they were three years old, with the peak incidence occurring at six to 18 months of age. 40% of them would have at least six recurrent episodes before the age of seven years.

Another crucially important public health aspect is that the disease burden and its complications have a much more significant impact on developing countries, with estimates by the World Health Organization (WHO) showing that approximately 51,000 deaths occur annually in children younger than five years, attributable to complications of AOM, such as intracranial infections. Additionally, approximately 60% of the hundreds of millions of individuals who develop chronic suppurative otitis manifest hearing impairment and behavioral changes.^1^

These AOM characteristics make the possibility of preventing it, through vaccines, the main and most attractive option for its management. Several studies have shown that the most common bacterial pathogens to cause AOM are *Streptococcus pneumoniae* together with nontypeable *Haemophilus influenzae* (NTHi) and *Moraxella catarrhalis* and to a lesser extent, *Streptococcus pyogenes* and *Staphylococcus aureus*. Finally, viral agents [influenza, respiratory syncytial virus (RSV), adenovirus, parainfluenza, and other viruses] account for approximately 30% of the cases and, together with bacteria, account for approximately 15% of cases of AOM.^2^

The incorporation of pneumococcal conjugate vaccines into routine infant immunization programs in an increasing number of countries worldwide, as well as the expansion of influenza vaccine recommendations for several pediatric age groups, has created an expectation of a decrease in AOM-related morbidity in these populations. The experience gained from the consolidation of these programs worldwide has taught us important lessons that deserve to be analyzed in this editorial.

The evaluation of the influenza vaccine impact on the reduction of AOM episodes in randomized controlled trials has shown conflicting results regarding the influenza vaccine efficacy in preventing AOM episodes in children. The presence of a broad spectrum of respiratory viruses potentially implicated in AOM in children, as well as the lower efficacy of influenza vaccine in infants – the age group most often affected by otitis – are the main reasons for the limited effectiveness of influenza vaccines in preventing AOM in children.^3^

Although they were developed essentially aiming at preventing invasive pneumococcal diseases (meningitis, sepsis, and bacteremic pneumonia), the contribution of pneumococcal conjugate vaccines (PCVs) to the reduction of mucosal infections (especially otitis) has always been a matter of debate. The results of the pre-licensing studies of these vaccines indicated a potential reduction of a maximum of 10% of AOM episodes among vaccinated children. Among the main reasons for the limited performance of PCVs in the prevention of AOM, we highlight the fact that the serotypes included in the several existing vaccines (CPV7, CPV10 and CPV13) are responsible for a limited proportion of these cases, considering the total number of circulating pneumococcal serotypes (at least 98 have been identified to date). Additionally, studies have shown a trend toward replacement of the pathogens causing AOM in vaccinated populations, with an increased prevalence of other otopathogens and pneumococcal serotypes not included in the vaccines (the replacement phenomenon).^4^ Moreover, these studies have shown a greater efficacy for outcomes considered more severe, associated with complex otitis, tympanostomy tube placement, and recurrent otitis media. These results suggested that the use of PCVs in young infants would be more effective for the prevention of complex otitis and chronic otitis with effusion than for prevention of episodes of simple acute otitis media.

*S. pneumoniae* is recognized as a pathogen responsible for early infections and can act as a trigger for middle ear lesions, generating a cascade of events that will result in subsequent polymicrobial infections, more often related to NTHi strains and biofilm formation. Infections caused by NTHi show greater risk of treatment failure and complications, such as recurrence and chronicity.^5^

Another important finding of these studies was that the late administration of PCVs in toddlers older than 1 year of age was not as effective in preventing complex cases, when compared to the administration of PCVs in young infants. The probable interpretation of this finding is that immunizing older children that have already experienced an initial episode of pneumococcal otitis cannot exert a protective effect in preventing the events that will lead to the formation of complex and chronic otitis media, since the pathogenic alterations that predispose these children to subsequent episodes have already been triggered and, at this stage, the serotypes included in the PCVs are not a relevant cause of infection.^5^

After the inclusion of PCVs into infant immunization programs, real-world observational studies found a significant reduction not only in AOM episodes, but mainly in the incidence and prevalence of complex cases. Overall, the magnitude of the reduction substantially exceeded the predictions based on the results obtained in the pre-licensing studies. For instance, in the United States, a few years after the introduction of PCV7, there was a reduction of up to 28% in frequent otitis media, 23% in ventilation tube placement rates, 42% in antimicrobial prescriptions and 43% in the rates of outpatient visits related to otitis media in children younger than 2 years. In the United Kingdom, there was a 22% reduction in the incidence rates of otitis media in children younger than 10 years of age following the introduction of PCV7, with an additional 19% reduction after PCV7 was replaced by PCV13.^4^, ^5^ Similarly, in Brazil, the rates of outpatient visits related to all-cause otitis AOM decreased 44% in children aged 2–23 months after the introduction of PCV10.^6^ Also worth mentioning is the reduction in the incidence rates of penicillin-resistant pneumococci, both among invasive disease isolates and middle ear and nasopharyngeal isolates.^4^, ^5^

The licensing of the 10-valent conjugate pneumococcal vaccine (PCV10), which utilizes *H. influenzae* D protein as carrier in 8 of its 10 serotypes, brought the perspective of a potential additional benefit of protection against infections caused by NTHi. However, to date it has not been possible to convincingly demonstrate, in the several countries where this vaccine was introduced, that this effect can be attained in children vaccinated with PCV10.

Therefore, despite the promising data regarding the impact of PCVs on the burden of AOM and its complications, it is important to note that the main benefit of these vaccines remains the prevention of invasive pneumococcal diseases in childhood. The development, in the coming decades, of more effective vaccines against the influenza virus, of vaccines against other clinically relevant viruses such as RSV, adenovirus and parainfluenza virus, of protein vaccines that can provide universal protection against all pneumococcal serotypes, as well as effective vaccines against NTHi infections, usually associated with recurrent and complex otitis, will be of crucial importance in ultimately considering otitis a vaccine-preventable disease.

## Conflicts of interest

The authors declare no conflicts of interest.
